# Dual-Action Grouper Bone and Wakame Hydrolysates Supplement Enhances Exercise Performance and Modulates Gut Microbiota in Mice

**DOI:** 10.3390/nu17182933

**Published:** 2025-09-11

**Authors:** Huey-Jine Chai, Tsung-Kai Yi, Yi-Feng Kao, Te-Hua Liu, Tsung-Yu Tsai, Yi-Ming Chen

**Affiliations:** 1 Fisheries Research Institute, Ministry of Agriculture, Keelung 202008, Taiwan; hjchai@mail.tfrin.gov.tw (H.-J.C.); tkyi@mail.tfrin.gov.tw (T.-K.Y.); yfkao@mail.tfrin.gov.tw (Y.-F.K.); 2 Department of Food Science, Fu Jen Catholic University, New Taipei City 242062, Taiwan; 147848@mail.fju.edu.tw (T.-H.L.); tytsai@mail.fju.edu.tw (T.-Y.T.); 3 Ph.D. Program in Nutrition and Food Science, Fu Jen Catholic University, New Taipei City 242062, Taiwan

**Keywords:** *Undaria pinnatifida*, grouper bone, gut microbiota, microbiome modulation, exercise performance

## Abstract

**Background**: Sustainable, dual-action ergogenic strategies are underexplored; most products target a single pathway and rarely upcycle seafood sidestreams. We therefore tested an upcycled formulation combining grouper bone hydrolysate and *Undaria pinnatifida* extract (GU) for ergogenic and microbiota effects in mice. We tested the ergogenic and microbiota modulating effects of GU in mice versus a vehicle and a BCAA control. **Methods**: GU was prepared via enzymatic hydrolysis of marine by-products and administered to male ICR mice for 4 weeks. Mice were divided into five groups (*n* = 7/group), receiving a vehicle control, a branched-chain amino acid (BCAA) supplement, or GU at three dose levels (1X, 2X, 3X) based on human-equivalent conversion. Exercise performance was assessed via grip strength and treadmill tests. Biochemical markers of fatigue, body composition, and safety indicators were also analyzed. Gut microbiota was evaluated using 16S rRNA sequencing and constrained principal coordinates analysis (CPCoA). **Results**: Four weeks of GU supplementation significantly enhanced exercise performance [(treadmill time ↑ Δ = 10.2–11.7 min versus vehicle (*q* ≤ 0.0002), grip strength ↑ Δ = 40.4–48.5 g (*q* ≤ 0.05)] and lean body mass [FFM ↑ at GU-1X (Δ = +0.80%, *q* = 0.0123)], surpassing the commercial BCAA control. Biochemical analyses indicated reduced exercise-induced lactate accumulation [(post-exercise lactate ↓ Δ = −2.71/−2.18 mmol·L^−1^, *q* = 0.0006)]. Gut microbiota profiling revealed distinct shifts in community composition in GU-treated groups, notably with an increased abundance of beneficial taxa such as *Lactobacillus* and *Muribaculum*. These alterations reflect the prebiotic activity of seaweed-derived polysaccharides, promoting a healthier gut microbial profile. Notably, GU improved metabolic markers (aspartate aminotransferase, [AST]; lactate dehydrogenase, [LDH]) without inducing toxicity. **Conclusions**: These findings indicate that GU functions as a dual-action supplement, coupling amino acid-mediated muscle anabolism with microbiome modulation to enhance physical performance and metabolic health. As an upcycled marine product, it presents a sustainable and effective strategy for exercise support. Future studies should include 90-day safety, mechanistic assays, and a preregistered human pilot.

## 1. Introduction

We used mice to precisely control dose/timing, apply standardized exercise assays, and collect invasive readouts; key muscle and gut–muscle pathways are conserved, enabling hypothesis-generating evaluation under tightly controlled conditions. For translation, doses were mapped by body surface area and findings are treated as exploratory effect-size estimates to inform a preregistered human pilot. Wakame (*Undaria pinnatifida*) contributes fucoidan (≈>150–300+ kDa), ~15–26% sulfated polysaccharide with microbiome-modulatory/prebiotic activity, which complements fish-bone peptides/minerals via the gut–metabolic axis.

Wakame (*Undaria pinnatifida*) is increasingly recognized as a dietary prebiotic: its sulfated polysaccharide fucoidan resists upper-gut digestion and is selectively fermented by colonic microbes, enriching *Bifidobacterium* and *Lactobacillus*, increasing butyrate production, and improving host metabolic markers [1]. Dietary wakame has been shown to enrich beneficial gut taxa such as *Bifidobacterium*, whereas fucoidan isolated from *U. pinnatifida* improves running endurance and lean muscle mass in mice and also augments intestinal *Lactobacillus* abundance [2].

Food-processing by-products pose a significant sustainability challenge, highlighting the need for innovative up-cycling approaches that transform waste streams into value-added products [3]. In the fisheries sector, nutrient-rich residues, such as fish bones, are still routinely discarded, imposing an avoidable environmental burden [4]. Likewise, primary processing of the edible seaweed *U. pinnatifida* (wakame) leaves behind substantial residual biomass with considerable nutritive potential. Redirecting these materials through upcycling exemplifies circular economy practices by simultaneously curbing waste generation and creating new functional foods [3,5].

Branched-chain amino acids (BCAAs)—leucine, isoleucine, and valine—play a central role in stimulating skeletal muscle protein synthesis and reducing both central and peripheral fatigue during exercise [6]. Randomized trials have shown that BCAA supplementation enhances endurance capacity and muscular strength in trained athletes [7] while also limiting exercise-induced muscle damage [8].

Wakame provides polysaccharides, particularly fucoidan, along with essential minerals and moderate protein content, contributing to documented health benefits such as prebiotic modulation of the gut microbiota [9]. Dietary wakame has been shown to enrich beneficial gut taxa such as *Bifidobacterium*, while fucoidan isolated from *U. pinnatifida* improves running endurance and lean muscle mass in mice, alongside increased intestinal *Lactobacillus* abundance [10].

Combining wakame-derived co-products with grouper bone hydrolysate (GBH) may thus deliver complementary benefits: BCAA-rich peptides to support muscle metabolism and fucoidan-rich polysaccharides to promote gut health, potentially producing additive or synergistic ergogenic effects.

In this study, we developed a sport nutrition supplement derived from hydrolyzed grouper bone and *U. pinnatifida* hydrolysate (GU) and evaluated its in vivo effects on exercise performance, fatigue-related biochemical markers, energy reserves, body composition, safety, and gut microbiota diversity in mice. The findings aim to demonstrate a sustainable strategy for enhancing physical performance using upcycled marine resources.

## 2. Materials and Methods

### 2.1. Supplement Preparation

Grouper fish bones (from *Epinephelus* spp. processing waste) were cleaned, crushed, and enzymatically hydrolyzed to produce a protein hydrolysate. Briefly, bones were incubated with a food-grade enzyme at 50 °C for 4 h (pH adjusted to 8), followed by heat treatment to inactivate the enzyme. The hydrolysate was filtered to remove insoluble minerals, and the peptide-rich filtrate was spray-dried to yield GBH powder [9].

Dried wakame seaweed (*U. pinnatifida*) was milled into a fine powder and similarly incubated with a food-grade enzyme at 50 °C for 4 h (at pH 8) then heat-treated for enzyme inactivation. The sports nutritional supplement was formulated by mixing GBH and GU powder at a 1:1 weight ratio, selected to ensure high BCAA and peptide content from GBH alongside dietary fibers and micronutrients from seaweed. The final supplement contained approximately 73.89 mg/g of fucose and 100 mg/g of BCAAs, based on the total dried weight. The mixed powder was stored at −20 °C until use.

### 2.2. Animals and Experimental Design

Male ICR mice (6 weeks old) were obtained from a certified breeding facility (BioLASCO, Taipei City, Taiwan) and housed in a controlled environment maintained at 24 ± 2 °C) and 60 ± 5% humidity, with a 12 h light/dark cycle (lights on from 8:00 to 20:00). Mice were provided standard laboratory chow (5001, PMI Feeds) and water ad libitum.

After a 2-week acclimatization period, 35 mice were randomly assigned to five groups (*n* = 7 per group). All animal procedures were approved by the Institutional Animal Care and Use Committee (IACUC) of Fu Jen Catholic University (New Taipei City, Taiwan; protocol A11305; approval date: 28 March 2024) and were conducted in accordance with its guidelines.

The GU dose for humans is typically 10 g/day. The equivalent mouse dose (1X = 2050 mg/kg) we calculated using the FDA recommended body surface area conversion method. Assuming a human weight of 60 kg, the human-equivalent dose (HED) is 10,000 mg/60 kg = 166.7 mg/kg; using a conversion factor of 12.3 (to account for body surface area differences between humans and mice), the mouse dose is 166.7 × 12.3 = 2050 mg/kg.

A total of 35 mice were randomly assigned to 5 groups (7 mice/group) for daily vehicle/GU oral gavage for 4 weeks. The 5 groups were vehicle control, positive control (PC; commercial BCCAs at 2050 mg/kg/day), GU-1X (2050 mg/kg/day), GU-2X (4100 mg/kg/day), and GU-3X (6150 mg/kg/day). The vehicle group received an equivalent volume and caloric content of the solution base. The PC group received a commercial BCAAs product at 2050 mg/kg/day, equivalent to a 60 kg human dose of 10 g/day.

### 2.3. Exercise Performance Assessments

On day 27 of the supplementation period, 30 min after GU administration, forelimb grip strength was assessed using a low-force testing system (PicoScope 2000, Pico Technology Limited, St. Neots, UK). Each mouse was allowed to grasp a metal bar with its forepaws and was gently pulled backward until it released the bar; the peak force (g) was recorded. Each measurement was repeated 10 times per mouse, and the highest consistent value was recorded as the grip strength. Endurance capacity was evaluated using a treadmill test, following the protocol described in a previous study [9].

### 2.4. Fatigue Biochemical Markers

After 28 days of GU supplementation, mice underwent a 15 min bout of moderate swimming in a water tank maintained at 30 °C and 65 cm in depth. Blood samples were collected immediately after exercise and centrifuged at 1500× *g* for 10 min at 4 °C. Serum was collected for biochemical analysis. Lactate, ammonia, and glucose levels were measured using an automated clinical chemistry analyzer (Hitachi 7060, Tokyo, Japan) [11].

### 2.5. Body Composition and Safety Evaluations

On day 29, whole-body composition was measured using time-domain nuclear magnetic resonance (TD-NMR) with an LF50 Minispec analyzer (Bruker, Karlsruhe, Germany). Mice were gently immobilized in 50 mm diameter plastic restraint tubes and scanned for 2 min. TD-NMR detects radio-frequency signals from hydrogen nuclei and utilizes tissue-specific relaxation constants (T_1_/T_2_) to differentiate and quantify fat mass (FM) and fat-free mass (FFM).

For safety assessment, mice were euthanized by CO_2_ asphyxiation, and major organs (liver, heart, lung, kidneys, epididymal fat pads [EFP], and muscles) were examined grossly and fixed in 10% neutral-buffered formalin for histopathological analysis. Tissues were sectioned and stained with hematoxylin and eosin (H&E), then examined microscopically by a veterinary pathologist blinded to treatment groups.

Blood was collected via cardiac puncture at the time of sacrifice for clinical biochemistry. Serum levels of alanine aminotransferase (ALT), aspartate aminotransferase (AST), lactate dehydrogenase (LDH), creatinine, creatine kinase (CK), total cholesterol (TC), triacylglycerol (TG), high-density lipoprotein (HDL), low-density lipoprotein (LDL), total protein (TP), albumin, and glucose were assessed using an autoanalyzer (Hitachi 7060, Tokyo, Japan) using standard reagents. Any signs of toxicity, such as organ inflammation, necrosis, or abnormal enzyme levels, were documented.

### 2.6. Gut Microbiota Analysis

To investigate the effects on gut microbiota, cecal samples were collected from each mouse immediately after euthanasia via CO_2_ asphyxiation and promptly frozen at −80 °C. Microbial DNA was extracted from feces using a commercial stool DNA kit (Qiagen, Hilden, Germany). The V3–V4 region of the 16S rRNA gene was amplified by PCR using universal primers and sequenced on an Illumina MiSeq platform, generating 2 × 300 bp paired-end reads [12,13,14].

Sequencing data were processed using the QIIME2 pipeline: reads were quality-filtered, denoised, and amplicon sequence variants (ASVs) were assigned taxonomic identities based on the SILVA 16S rRNA reference database [15]. Constrained Principal Coordinates Analysis (CPCoA), an ordination method that combines PCoA with Redundancy Analysis, was used to visualize differences in microbial community composition. CPCoA identifies axes that maximize variation among predefined groups, enabling simultaneous evaluation of sample clustering and the influence of experimental variables. A minimum of three groups is required for CPCoA [16,17]. Relative abundances of bacterial taxa were calculated at the phylum, family, and genus levels for each group. For the microbiome subset, each treatment group was housed in a single cage; thus, the cage was identical to the group and could not be modeled as a random effect nor used to constrain permutations. Alpha diversity (Shannon, Simpson, Pielou, Faith’s PD, Menhinick, Margalef) and sequencing depth/rarefaction, negatives and feature filtering, and PERMDISP are reported. Beta-diversity ordinations and PERMANOVA (unconstrained) are presented descriptively.

### 2.7. Statistical Analysis

Continuous variables are summarized as mean ± standard deviation (SD). Between-group comparisons used one-way ANOVA with Tukey’s honestly significant difference post hoc test; two-sided *p* < 0.05 was considered statistically significant. Analyses were conducted in SAS 9.4 (SAS Institute Inc., Cary, NC, USA).

Within an effect-size-first framework, we prioritize point estimates and 95% confidence intervals (CIs), with two-sided *p*-values as secondary evidence. The prespecified primary endpoint (Week-4 treadmill time) was analyzed by ANCOVA adjusting for baseline. Continuous outcomes are presented as absolute mean differences (Δ) with 95% CIs; positively skewed biomarkers were log-transformed and summarized as geometric mean ratios (GMRs) with 95% CIs. Multiplicity was controlled within outcome families (Performance, Body composition, Biochemistry, Acute exercise biochemistry, Tissue weight) using the Benjamini–Hochberg false discovery rate (FDR) at *q* = 0.05; for single-hypothesis families, the adjusted q equals the nominal two-sided *p* value. Standardized effect sizes (Hedges g) are reported and interpreted as trivial (<0.20), small (0.20–0.49), moderate (0.50–0.79), and large (≥0.80).

## 3. Results

### 3.1. General Observations and Body Weight

**Figure 1:**
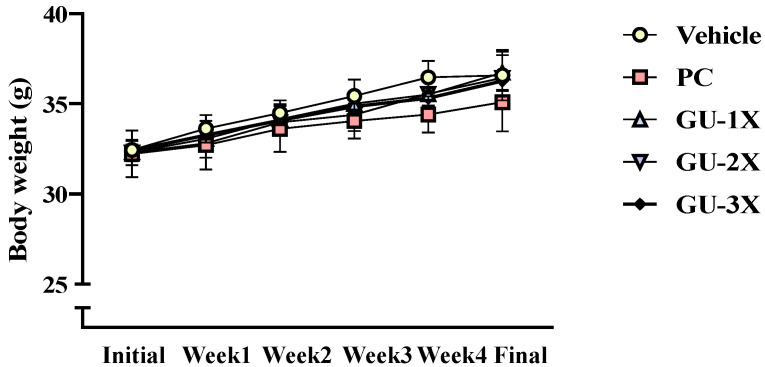
Time course of body weight changes during the 4-week intervention. Male ICR mice (*n* = 7 per group) were orally administered either vehicle, a commercial BCAA-positive control (PC), or the grouper bone/U. pinnatifida complex (GU-1X, GU-2X, or GU-3X) daily for 4 weeks. Body weight was measured at baseline and weekly thereafter, with a final measurement taken prior to euthanasia. Values are presented as mean ± SD.

All mice remained healthy throughout the 4-week study period, with no abnormal behaviors or signs of toxicity observed in any group. Body weight increased steadily with age in all groups (Figure 1), and there were no significant differences in weekly body weights or total weight gain among the vehicle and GU-treated groups (*p* > 0.05).

After 4 weeks, final body weights were as follows: 35.59 ± 1.40 g (vehicle), 35.09 ± 1.60 g (PC), 36.58 ± 1.19 g (GU-1X), 36.44 ± 1.45 g (GU-2X), and 36.27 ± 0.53 g (GU-3X), with no statistically significant differences among groups (*p* = 0.1138). Food and water intake were also comparable across all groups (diet intake, *p* = 0.9930; water intake, *p* = 0.4142) (Table 1). Absolute hindlimb muscle mass increased significantly compared to the vehicle group: the PC group (Δ = 0.06, *p* = 0.0064), GU-1X (Δ = 0.08 g, CI = 0.34–0.42, ES = 0.0015, *p* = 0.0001, *q* = 0.0002), GU-2X (Δ = 0.09 g, CI = 0.34–0.40, ES = 2.85, *p* = 0.0006, *q* = 0.0006), and GU-3X (Δ = 0.11 g, CI = 0.37–0.40, ES = 0.32, *p* < 0.001, *q* ≤ 0.0002). When normalized to body weight, relative muscle mass was 0.25% to 0.32% higher in all GU-supplemented groups (Δ = 0.25–0.32%, CI = 0.92–1.23, ES = 2.39–2.65, all *q* < 0.05 for each comparison). EFP mass and major organ weights remained unchanged, indicating that lean mass gains occurred without concurrent increases in adiposity or organ hypertrophy.

**Figure 2:**
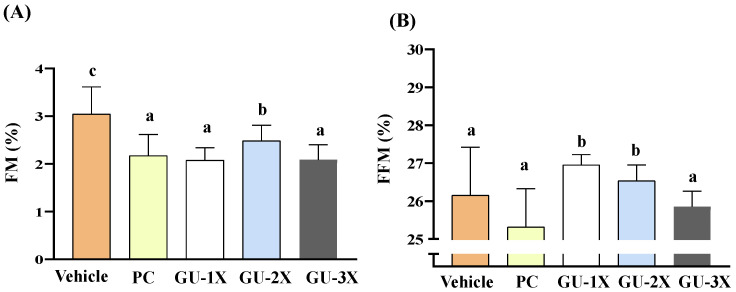
Effect of GU supplementation on body composition. (**A**) Fat mass percentage and (**B**) free-fat mass percentage in male ICR mice after 4 weeks of treatment with vehicle (glucose water), PC (2050 mg/kg/day commercial BCAAs), GU-1X (2050 mg/kg/day GU), GU-2X (4100 mg/kg/day GU), or GU-3X (6150 mg/kg/day GU). Mice underwent an exercise performance test 1 h after the administered dose. Different letters (a, b, c) indicate statistically significant differences between groups (*p* < 0.05).

Collectively, these findings indicate that GU supplementation selectively promotes muscle accretion without affecting appetite, overall growth, or visceral organ integrity, supporting its potential as a safe and effective ergogenic aid. To further evaluate changes in body composition, we conducted time-domain nuclear magnetic resonance (TD-NMR) analysis. Significant decreases in fat mass (FM) were observed in GU-1X (Δ = −0.33%, CI = 1.92–2.23, ES = 0.74, *p* < 0.0001, *q* ≤ 0.0006), GU-2X (Δ = −0.32%, CI = 2.30–2.67, ES = 0.71, *p* = 0.0005, *q* = 0.0015), and GU-3X (Δ = −0.53%, CI = 1.90–2.27, ES = 1.15, *p* < 0.0001, *q* = 0.0060) compared to the vehicle group (Figure 2A). Conversely, free-fat mass (FFM) was significantly increased in the GU-1X (Δ = 0.80%, CI = 26.8–27.1, ES = 0.87, *p* = 0.0082, *q* = 0.0123) *(*Figure 2B).

### 3.2. Muscle Strength and Endurance Performance

**Figure 3:**
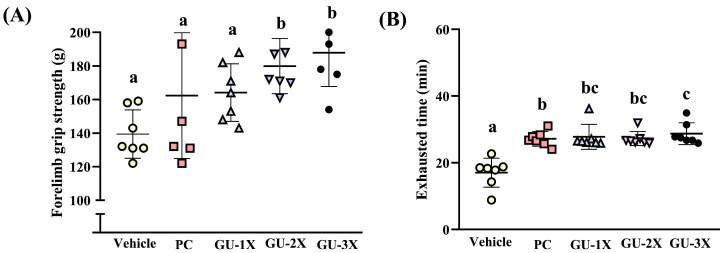
Effect of GU supplementation on exercise performance: forelimb grip strength (**A**) and treadmill exhausted time (**B**). Male ICR mice were pretreated for 4 weeks with vehicle (glucose water), PC (2050 mg/kg/day commercial BCAAs), GU-1X (2050 mg/kg/day GU), GU-2X (4100 mg/kg/day GU), or GU-3X (6150 mg/kg/day GU). Exercise performance tests were conducted 1 h after the administered dose. Different letters (a, b, c) indicate statistically significant differences between groups (*p* < 0.05).

As shown in Figure 3A, the grip strength values for the vehicle, PC, GU-1X, GU-2X, and GU-3X groups were 139.4 ± 14.4, 162.3 ± 37.5, 164.1 ± 17.2, 179.9 ± 16.4, and 187.9 ± 20.1 g, respectively. Grip strength was significantly higher in the GU-2X (Δ = 40.43 g, CI = 164–195, ES = 2.62, *p* = 0.0023, *q* ≤ 0.0028), and GU-3X (Δ = 48.53 g, CI = 169–207, ES = 2.77, *p* = 0.0004, *q* ≤ 0.0507) groups compared to the vehicle group and, respectively. Endurance performance, assessed by an exhaustive treadmill test, was also improved by supplementation. The treadmill running times for the vehicle, PC, GU-1X, GU-2X, and GU-3X groups were 17.0 ± 4.4, 27.2 ± 2.2, 27.8 ± 3.7, 27.3 ± 2.1, and 28.7 ± 3.3 min, respectively. Compared with the vehicle group, GU-1X (Δ = 10.74 min, CI = 24.12–31.33, ES = 2.66, *p* < 0.0001, *q* ≤ 0.0002), GU-2X (Δ = 10.23 min, CI = 25.24–29.30, ES = 2.99, *p* < 0.0001, *q* ≤ 0.0002), and GU-3X (Δ = 11.68 min, CI = 25.76–31.68, ES = 3.01, *p* < 0.0001 *q* ≤ 0.0002) showed significantly longer running times, respectively (Figure 3B).

Both PC and GU significantly enhanced grip strength and endurance relative to the vehicle. Taken together, GU supplementation demonstrated superior effects on both power-related (grip strength) and endurance-related (treadmill running) performance, matching or exceeding the effects of commercial BCAAs and supporting its potential as a broad-spectrum ergogenic aid.

### 3.3. Blood Biochemical Markers of Fatigue

**Figure 4:**
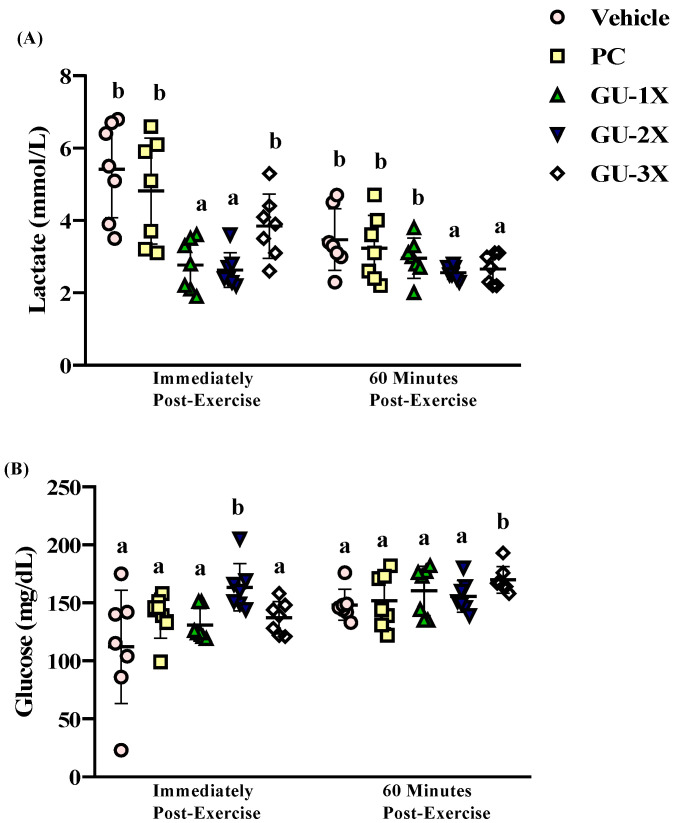
Effect of GU supplementation on acute exercise-induced biochemical markers. (**A**) Serum lactate levels; (**B**) serum glucose levels. Male ICR mice were pretreated for 4 weeks with vehicle (glucose water), PC (2050 mg/kg/day commercial BCAAs), GU-1X (2050 mg/kg/day GU), GU-2X (4100 mg/kg/day GU), or GU-3X (6150mg/kg/day GU). Mice underwent exercise performance testing 1 h after the administered dose. Different letters (a, b) indicate statistically significant differences between groups (*p* < 0.05).

After 15 min exercise, serum lactate levels in the vehicle, PC, GU-1X, GU-2X, and GU-3X groups were 5.4 ± 1.3, 4.8 ± 1.5, 2.7 ± 0.7, 3.2 ± 0.5, and 3.8 ± 0.9 mmol/L, respectively. Lactate levels were significantly lower in the GU-1X (Δ = −2.71 mmol/L, CI = 2.17–3.37, ES = 2.59, *p* < 0.0001, *q* = 0.0006) and GU-2X (Δ = −2.18 mmol/L, CI = 2.18–3.08, ES = 2.23, *p* < 0.0001, *q* = 0.0006) groups compared to the vehicle (Figure 4A). After 60 min rest, lactate levels remained significantly reduced in the GU-2X (Δ = −0.91 mmol/L, CI = 2.38–2.74, ES = 1.54, *p* = 0.0127, *q* = 0.0304) and GU-3X (Δ = −0.81 mmol/L, CI = 2.27–3.05, ES = 1.26, *p* = 0.0250, *q* = 0.0453) groups (Figure 4A).

Post-exercise serum glucose levels were elevated in all GU-treated groups, with GU-2X reaching 164 ± 20 mg/dL—increase relative to vehicle (Δ = 51.17 mg/dL, CI = 144.63–182.23, ES = 1.39, *p* = 0.0012, *q* = 0.0048) (Figure 4B). After 60 min of rest, glucose levels remained significantly elevated in the GU-3X group (Δ = 21.60 mg/dL, CI = 159.49–180.23, ES = 1.83, *p* = 0.0264, *q* = 0.0453) higher than vehicle (Figure 4B). These findings indicate that GU supplementation modulates acute fatigue biomarkers by reducing lactate accumulation and promoting faster post-exercise recovery. The elevated glucose level, particularly in GU-3X, may reflect enhanced hepatic glucose output or glycogen sparing, contributing to sustained energy availability. Combined with the lack of changes in organ or EFP weights, these effects support the conclusion that GU supplementation alleviates metabolic fatigue without systemic adverse effects, thereby enhancing both muscular strength and endurance.

### 3.4. Body Composition and Organ Health

**Figure 5:**
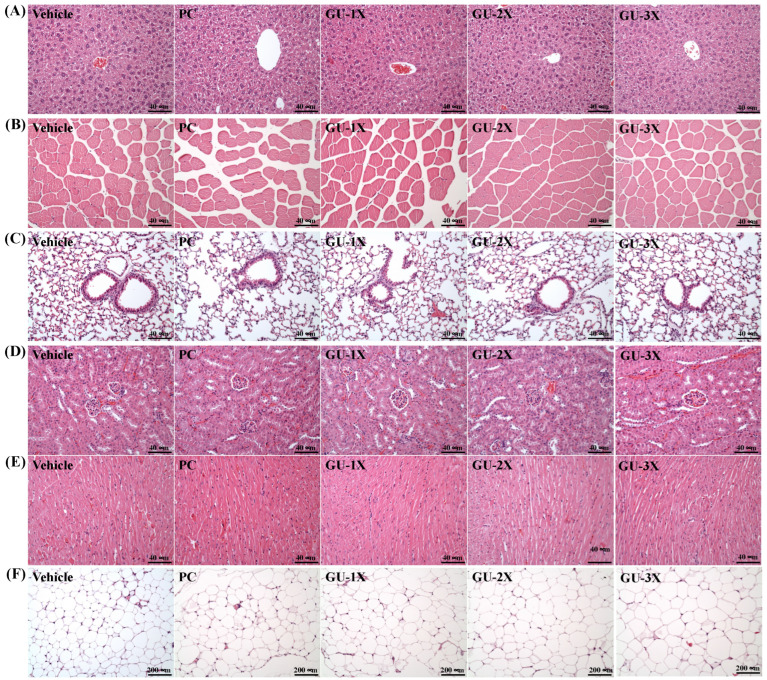
Effect of GU supplementation on tissue histology. Representative histological images of the liver (**A**), skeletal muscle (**B**), lung (**C**), kidney (**D**), heart (**E**), and EFP (**F**). Tissue sections were stained with hematoxylin and eosin (H&E) and observed under light microscopy at 200× magnification (**A**–**F**). EFP: epididymal fat pad.

Serum clinical chemistry data are summarized in Table 2. Compared to the vehicle group, GU supplementation significantly reduced hepatic enzymes AST and LDH (ES = 1.41–1.67, *p* < 0.05, *q* < 0.05), indicating decreased hepatocellular stress. Serum creatinine, a marker of renal function, was also significantly lower in both the PC and all GU groups (ES = 1.57–1.96, *p* < 0.05, *q* < 0.05), suggesting preserved or improved kidney health. No significant differences were observed among groups for ALT, total cholesterol, HDL-C, LDL-C, triacylglycerols, total protein, albumin, CK, or glucose (all *p* > 0.05). All measured values remained within normal physiological ranges, demonstrating the supplement was well tolerated and did not adversely affect hepatic, renal, or metabolic function. Histological analysis revealed no signs of hepatic inflammation, steatosis, or necrosis in any group. Renal glomeruli and tubules appeared intact, and cardiac tissue showed no inflammation or cellular infiltration (Figure 5). Collectively, these findings confirm the safety of the fish bone/wakame (GU) supplement at the tested doses, demonstrating that the improved physical performance occurred without signs of systemic toxicity.

### 3.5. Analysis of 16S rRNA Gene Sequences Following 4-Week GU Supplementation

Alpha diversity did not differ among groups (Shannon, Simpson, Pielou, Faith’s PD, Menhinick, Margalef; all q_FDR ≥ 0.05); effect-size estimates with 95% CIs were near zero.

**Figure 6:**
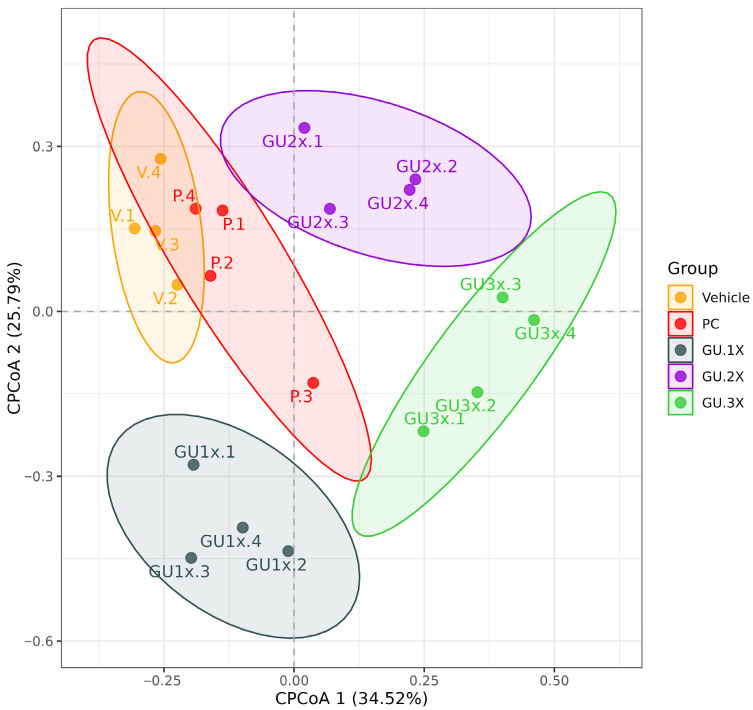
The CPCoA plot visualizes the dissimilarities in microbial community composition across groups: vehicle, PC, GU-1X, GU-2X, and GU-3X. Each point represents an individual sample, with group-specific colors. Ellipses denote the 95% confidence interval for each group. The x-axis (CPCoA1) and y-axis (CPCoA2) explain 34.52% and 25.79% of the total variance, respectively. The spatial separation among groups indicates differences in gut microbial community structure in response to treatment.

Beta-diversity analysis using PCoA revealed distinct separation between the microbiota profiles of GU-supplemented mice and the vehicle group, indicating differences in overall community composition. Canonical PCoA (CPCoA) further demonstrated clear clustering of microbial communities across treatment groups (Figure 6). The first and second canonical coordinates (CPCoA1 and CPCoA2) accounted for 34.52% and 25.79% of the total variance, respectively. Notably, samples from the GU-3X group formed a distinct cluster, separate from the vehicle, PC, and GU groups, suggesting a potential dose-dependent effect of GU supplementation on gut microbial composition.

This pattern was supported by PERMANOVA results (Table 3), which quantified the extent of variation in microbial community composition attributable to treatment. Among all comparisons, the GU-3X group showed a statistically significant difference from the vehicle group (*F* = 0.7506, *R*^2^ = 0.2259, *p* = 0.0210), indicating that 22.59% of the variance in microbial composition could be explained by this treatment. In contrast, comparisons between the vehicle group and PC (*p* = 1.000), GU-1X (*p* = 0.1880), and GU-2X (*p* = 0.3660) were not statistically significant, although *R*^2^ values showed a modest upward trend with increasing GU dose.

**Figure 7:**
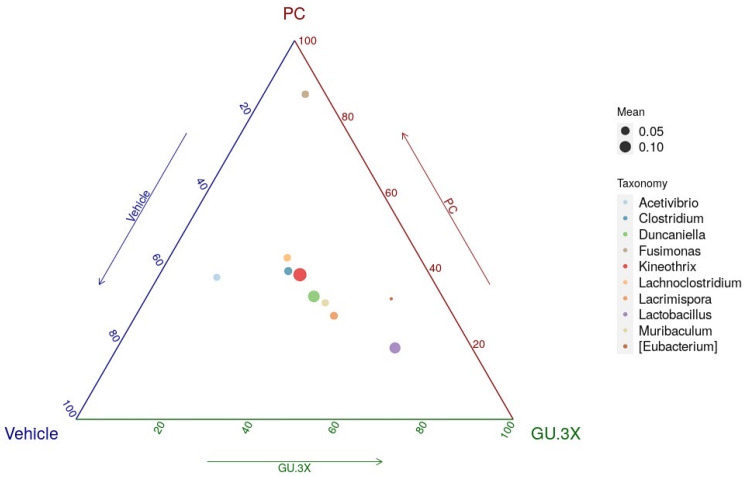
Ternary plot illustrating the distribution of bacterial genera among three treatment groups.

To further characterize group-specific microbial features, a ternary plot was generated to visualize the relative abundance distribution of key bacterial genera among the vehicle, PC, and GU-3X groups (Figure 7). In the plot, each vertex of the triangle represents one group, and each colored circle corresponds to a specific genus. The proximity of a circle to a given vertex indicates higher relative abundance of that genus in the corresponding group, while circle size reflects the mean abundance across all samples.

Figure 7 highlights distinct distribution patterns of bacterial genera among the vehicle, PC, and GU-3X groups. Notably, several genera exhibited group-enriched trends. *Fusimonas*, *Clostridium*, and *Lachnoclostridium* were positioned closer to the PC apex, suggesting greater abundance in the PC group compared to vehicle and GU-3X. In contrast, *Muribaculum* and *Lactobacillus* were located nearer the GU-3X vertex, indicating selective enrichment of these beneficial taxa in the GU-3X group. Meanwhile, *Acetivibrio* appeared modestly enriched in the vehicle group.

These distribution patterns suggest that GU-3X supplementation may promote the proliferation of specific microbial genera (e.g., *Lactobacillus*, *Muribaculum*), potentially contributing to the overall microbial shift observed in CPCoA and PERMANOVA analyses. Conversely, genera enriched in the PC group may represent less favorable microbial profiles. Overall, the ternary plot provides a visual summary of genus-level compositional differences across groups, highlighting key microbial targets influenced by GU-3X intervention.

Each vertex of the triangle represents one of the three groups: vehicle, PC, and GU-3X. Each circle corresponds to a bacterial genus, with colors indicating different taxa as shown in the legend. Circle size is proportional to the mean relative abundance of the genus across all samples. The position of each circle within the triangle reflects its compositional distribution among the groups, with genera located closer to a given vertex being relatively more abundant in that group. This ternary plot analysis was used to visualize compositional differences across the three groups.

## 4. Discussion

This study demonstrates the ergogenic efficacy of the GU in enhancing exercise performance and improving body composition. Mice supplemented with GU showed significantly increased muscle strength and endurance compared to the vehicle group, with effects comparable to or exceeding those of BCAA supplementation. These benefits align with the known role of BCAAs, particularly leucine, in promoting skeletal muscle protein synthesis through activation of the mTOR pathway [18]. Leucine acts as a key nutrient signal that triggers mTORC1 activation, thereby accelerating muscle protein synthesis and recovery [19,20]. Consistent with this mechanism, GU-treated mice displayed increased hindlimb muscle mass without corresponding fat accumulation, indicating a selective anabolic effect. Additionally, the high leucine and essential amino acid content of the fish bone hydrolysate likely contributed to this effect by providing both the substrates and anabolic signals required for muscle repair and growth [21]. Indeed, leucine-enriched nutrition has been shown to rapidly stimulate muscle protein synthesis and mitigate muscle wasting conditions [19,22]. Therefore, the BCAA-rich peptides in GU likely promoted skeletal muscle hypertrophy via mTOR-dependent anabolic signaling pathways.

The ergogenic effects of GU cannot be attributed solely to its BCAA content. The *U. pinnatifida*-derived components—particularly fucoidan polysaccharides and marine-derived minerals—likely contribute synergistically. Fucoidan extracted from *U. pinnatifida* has been reported to enhance endurance performance and promote muscle hypertrophy in murine models [2]. Although mitochondrial biogenesis was not directly assessed in this study, the improved treadmill performance observed in GU-treated mice suggests enhanced aerobic capacity, potentially mediated by increased mitochondrial density. Notably, GU-fed mice achieved endurance improvements comparable to those receiving pure BCAAs, indicating a possible additive or synergistic effect between fucoidan and BCAAs. Additionally, the antioxidant and anti-inflammatory properties of fucoidan may have mitigated exercise-induced oxidative stress, supporting recovery and overall performance [23].

Notably, GU supplementation led to favorable changes in body composition that were not fully replicated by BCAAs alone. Across all doses, GU significantly reduced adipose tissue percentage while increasing fat-free mass, whereas BCAAs did not significantly reduce fat mass. This indicates that other bioactive components in the fish/seaweed complex may promote nutrient repartitioning toward lean tissue accretion. Previous studies in metabolic syndrome models have indicated that fucoidan and other seaweed-derived compounds can enhance insulin sensitivity and lipid metabolism [22]. Moreover, the protein peptides and calcium/phosphate content from fish bone may have contributed to increases in lean and possible bone mass, although bone density was not specifically assessed in this study [9,24].

Notably, GU supplementation was well tolerated and safe. Treated mice showed no signs of toxicity, and several clinical chemistry markers improved. Notably, serum AST and LDH levels were significantly lower in GU-treated groups compared to the vehicle group. As elevated AST and LDH typically reflect tissue damage or hepatic stress, their reduction suggests decreased muscle damage and reduced metabolic strain during exercise. These findings are consistent with previous reports showing that BCAA supplementation can attenuate exercise-induced muscle damage, as indicated by lower CK and LDH levels in human studies [25]. Furthermore, reduced serum creatinine levels in GU groups may reflect improved renal-metabolic health or more efficient protein utilization. Collectively, these results highlight that GU enhances exercise performance and body composition without adverse effects, supporting its potential as a safe and effective ergogenic aid.

A major finding of this study is that GU supplementation markedly modulated the gut microbiota, suggesting a potent prebiotic effect attributable to the wakame components. After four weeks of intervention, 16S rDNA sequencing revealed distinct alternations in microbial community structure in GU-fed mice compared to the vehicle group. Notably, the GU-3X group showed a gut microbiota profile that clearly diverged from both vehicle and BCAA-only groups on PCoA. Taxonomic analysis indicated an enrichment of beneficial bacterial genera, particularly *Lactobacillus* and *Muribaculum*, in the GU-3X group, while the vehicle and BCAA groups exhibited higher relative abundances of potentially less favorable genera such as *Fusimonas*, *Clostridium*, and *Lachnoclostridium*. These findings suggest that the fiber rich wakame extract in GU selectively fostered a more favorable gut microbial environment, in contrast to the protein-focused BCAA supplement, which lacks fermentable fiber.

Many of the taxa promoted by GU are associated with gut health and metabolic benefits. *Lactobacillus* is a well-known probiotic genus that improve intestinal barrier function and modulate immunity [26]. The observed increase in *Lactobacillus* abundance in the GU-3X group supports the hypothesis that seaweed-derived polysaccharides act as prebiotics. Moreover, fucoidan and other seaweed fibers resist digestion in the upper gastrointestinal tract and are fermented by colonic microbes, promoting the selective proliferation of *Bifidobacterium* and *Lactobacillus* species [27]. Yoshinaga et al. (2018) [10] reported that dietary wakame intake in humans enriched *Bifidobacterium longum*, increased GLP-1 secretion, and improved metabolic parameters. Similarly, in rodent models, fucoidan from various brown seaweeds (including *Undaria*) has consistently been shown to increase *Lactobacillus* populations and short-chain fatty acids (SCFA)-producing taxa while reducing the abundance of potentially pathogenic bacteria [28,29]. Our findings align with these studies, indicating that the inclusion of *Undaria*-derived fucoidan and fibers in the GU supplement promoted a gut environment conducive to fermentative, beneficial microbes.

The increase in *Muribaculum* (family *Muribaculaceae*) observed in GU-supplemented mice is another noteworthy finding. *Muribaculaceae* are prominent fiber-degrading bacteria in the rodent gut and are considered indicators of a healthy, fiber-rich diet. These bacteria are typically abundant in lean mice fed standard chow and tend to decline in animals consuming high-fat or low-fiber diets [30]. *Muribaculaceae* are highly efficient at fermenting complex polysaccharides into SCFAs, particularly acetate and propionate. In a study by Cao et al. (2020), dietary enrichment with polyphenols and fiber in obese mice promoted the growth of *Muribaculaceae*, which was inversely correlated with adiposity, implicating a potential role in obesity prevention [31]. The elevation of *Muribaculum* in the GU-3X group aligns with our findings of reduced fat mass in these mice. Therefore, the enrichment of *Muribaculaceae* and *Lactobacillaceae* may have contributed to improved host energy metabolism, potentially through increased SCFA production and modulation of appetite, satiety, or energy expenditure [32].

Although fecal SCFAs were not measured in this study, previous in vitro research has shown that *Undaria* fucoidan can significantly increase butyrate production by gut microbiota [1]. An elegant example of the microbiota’s impact on exercise endurance was given by Scheiman et al. (2019), who observed that certain gut bacterial taxa metabolize lactate into propionate, thereby enhancing treadmill running time in mice [33]. In our study, the reduced serum lactate levels and improved performance observed in GU-treated mice may be partially attributable to a more favorable microbiome that rapidly metabolizes lactate (perhaps by *Lactobacillus*) and generates fatigue-alleviating compounds like propionate and butyrate.

Our data also suggest that GU supplementation modulated the gut microbiota in ways that may directly enhance exercise performance and metabolic health. The genus *Lactobacillus* has been linked to anti-fatigue effects in multiple studies, largely through mechanisms such as enhanced muscle glycogen storage, increased antioxidant capacity, and altered substrate utilization mediated via the gut–muscle axis [34]. In our study, although we administered a prebiotic rather than live probiotic bacteria, the net effect was an enrichment of *Lactobacilli* in the gut, functionally similar to probiotic supplementation. Therefore, it is reasonable to propose that the GU-induced expansion of *Lactobacillus* contributed to the improvements in endurance and post-exercise recovery in the mice.

The presence of *Lactobacillus* may have promoted glycogen sparing through enhanced butyrate production, providing an alternative energy source, and may have attenuated skeletal muscle inflammation via bioactive metabolites [35]. Despite receiving equivalent amounts of protein and energy, the BCAA-only group lacked fermentable substrates required to sustain beneficial microbial populations. This may account for their less favorable microbiota composition, including elevated levels of *Fusimonas*, a genus recently implicated in dysregulated lipid metabolism and metabolic disorders [36].

Additionally, microbial diversity in the GU groups appeared to increase, consistent with the effects commonly observed with dietary fiber supplementation. A diverse gut microbiota is generally more resilient and is associated with improved host health [37]. Notably, our constrained CPCoA showed that the GU-3X group formed a distinct cluster, indicating that a sufficiently high dose of the seaweed–fish complex can overcome individual variability and establish a consistent microbial community structure.

Our mouse results provide effect-size estimates in domains that map to human function: GU increased treadmill time-to-exhaustion, improved forelimb grip, reduced post-exercise lactate, and increased lean mass at GU-1X. These align with human constructs of endurance, maximal strength, recovery metabolism, and body composition. Translation will leverage body surface area mapping (mouse GU-1X = 2050 mg·kg^−1^ derived from a human serving) and the complementary biology of the two components: fish-bone hydrolysate support acute amino-nitrogen availability, while wakame fucoidan (low-MW, <500 Da; ~15–26% sulfation) is expected to act sub-acutely via the gut–metabolic axis. Accordingly, we outline a preregistered human pilot to test safety/PK/tolerability and exploratory performance endpoints (time-to-exhaustion, RPE, lactate kinetics), with stool microbiome and SCFAs as mechanistic readouts.

## 5. Conclusions

In summary, this study demonstrates that combining amino acids with fermentable seaweed-derived polysaccharides provides synergistic benefits consistent with a prebiotic model. Unlike BCAA supplementation alone, the GU formulation selectively enriched short-chain fatty acid-producing and anti-inflammatory taxa, including *Lactobacillus* and *Muribaculum*. These microbial shifts likely enhanced energy substrate availability, supported gut and muscle homeostasis, and contributed to the improvement in exercise capacity. These findings highlight GU as a promising prebiotic candidate for optimizing host–microbiome interactions to support endurance, recovery, and metabolic health. Future studies should validate these effects in human and clarify the role of microbial metabolites in mediating exercise adaptations.

Future work will confirm the subchronic safety and mechanisms (SCFAs, RER/VO_2_, muscle signaling) in multi-cage, sex-balanced, isonitrogenous 2 × 2 factorial designs. A preregistered human pilot (safety/PK/tolerability with exploratory performance and microbiome endpoints) will test translational potential.

Limitations: *n* = 7/group and 4-week exposure limit the detection of rare/delayed toxicities; groups were male ICR only, chow-fed and untrained. The microbiome subset had one cage per group, so cage and treatment were confounded and all 16S inferences are exploratory. We did not include isonitrogenous/isocaloric factorial arms (GBH-only, wakame-only), and mechanistic assays (SCFAs, RER/VO_2_, muscle signaling) were not collected. Dosing assessments focused on 30–60 min post-gavage, which captures peptide kinetics but may miss later fucoidan effects. We mitigate multiplicity with BH-FDR within outcome families but acknowledge residual multiplicity. To address these gaps, future studies will use multi-cage, sex-balanced designs, add isonitrogenous 2 × 2 factorial arms, incorporate mechanistic endpoints, and complete a GLP-aligned 90-day study before human trials.

## Figures and Tables

**Table 1:** General characteristics of mice supplemented with GU for 4 weeks.

Characteristics	Vehicle	PC	GU-1X	GU-2X	GU-3X
Initial BW (g)	32.44 ± 0.51	32.23 ± 1.30	32.30 ± 0.58	32.35 ± 0.38	32.42 ± 0.31
Final BW (g)	36.59 ± 1.40	35.09 ± 1.60	36.58 ± 1.19	36.44 ± 1.45	36.27 ± 0.53
Food intake (g/day)	7.96 ± 1.05	8.02 ± 0.27	8.05 ± 0.31	7.98 ± 0.70	8.02 ± 0.56
Water intake (mL/day)	5.92 ± 0.26	6.13 ± 0.15	6.16 ± 0.37	5.97 ± 0.37	5.99 ± 0.26
Liver (g)	1.46 ± 0.06	1.29 ± 0.10	1.32 ± 0.15	1.38 ± 0.11	1.34 ± 0.14
Kidney (g)	0.66 ± 0.05	0.68 ± 0.03	0.67 ± 0.04	0.67 ± 0.04	0.67 ± 0.04
EFP (g)	0.49 ± 0.08	0.43 ± 0.10	0.42 ± 0.06	0.42 ± 0.10	0.41 ± 0.07
Heart (g)	0.20 ± 0.01	0.18 ± 0.02	0.18 ± 0.01	0.19 ± 0.01	0.19 ± 0.01
Lung (g)	0.27 ± 0.01	0.28 ± 0.04	0.28 ± 0.01	0.26 ± 0.01	0.27 ± 0.01
Muscle (g)	0.30 ± 0.02 ^a^	0.35 ± 0.04 ^b^	0.38 ± 0.04 ^b^	0.39 ± 0.04 ^b^	0.40 ± 0.04 ^b^
Relative liver weight (%)	3.91 ± 0.23	3.65 ± 0.22	3.62 ± 0.32	3.69 ± 0.21	3.72 ± 0.33
Relative kidney weight (%)	1.94 ± 0.16	1.92 ± 0.08	1.87 ± 0.13	1.89 ± 0.07	1.95 ± 0.09
Relative EFP weight (%)	1.32 ± 0.19	1.21 ± 0.29	1.14 ± 0.18	1.13 ± 0.28	1.14 ± 0.19
Relative heart weight (%)	0.52 ± 0.04	0.50 ± 0.04	0.51 ± 0.03	0.51 ± 0.04	0.51 ± 0.04
Relative Lung weight (%)	0.76 ± 0.04	0.78 ± 0.10	0.78 ± 0.01	0.74 ± 0.01	0.75 ± 0.04
Relative Muscle weight (%)	0.79 ± 0.03 ^a^	1.00 ± 0.11 ^b^	1.04 ± 0.13 ^b^	1.07 ± 0.10 ^b^	1.11 ± 0.33 ^b^

Data is presented as mean ± SD (*n* = 7 mice per group). Different superscript letters (^a,b^) within the same row indicate significant differences (*p* < 0.05). Muscle mass includes both gastrocnemius and soleus muscles from the hind limbs. Mice received daily oral administration of vehicle (glucose water), positive control (PC; 2050 mg/kg/day commercial BCAAs), GU-1X (2050 mg/kg/day GU), GU-2X (4100 mg/kg/day GU), or GU-3X (6150 mg/kg/day GU). BW, body weight; EFP, epididymal fat pad.

**Table 2:** Effects of 4-week GU supplementation on biochemical parameters.

Parameter	Vehicle	PC	GU-1X	GU-2X	GU-3X
AST (U/L)	157 ± 61 ^b^	118 ± 47 ^b^	86 ± 22 ^a^	90 ± 11 ^a^	89 ± 20 ^a^
ALT (U/L)	108 ± 23	97 ± 37	95 ± 30	84 ± 24	75 ± 20
LDH (U/L)	633 ± 151 ^b^	436 ± 127 ^a^	434 ± 74 ^a^	445 ± 67 ^a^	455 ± 89 ^a^
Creatinine (mg/dL)	0.14 ± 0.03 ^b^	0.09 ± 0.02 ^a^	0.10 ± 0.02 ^a^	0.10 ± 0.02 ^a^	0.10 ± 0.02 ^a^
TC (mg/dL)	138 ± 24	140 ± 21	146 ± 21	144 ± 19	137 ± 22
HDL (mg/dL)	109 ± 13	105 ± 18	110 ± 14	110 ± 10	102 ± 15
LDL (mg/dL)	2.48 ± 0.71	2.20 ± 0.61	2.53 ± 1.02	2.60 ± 0.58	2.54 ± 0.30
TG (mg/dL)	185 ± 44	137 ± 32	179 ± 32	191 ± 31	196 ± 23
TP (g/dL)	5.07 ± 0.26	4.94 ± 0.15	5.02 ± 0.08	5.08 ± 0.09	5.04 ± 0.16
Albumin (g/dL)	2.80 ± 0.19	2.75 ± 0.16	2.82 ± 0.08	2.80 ± 0.07	2.80 ± 0.06
CK (U/L)	216 ± 93	257 ± 112	160 ± 49	198 ± 31	222 ± 92
Glucose (mg/dL)	196 ± 17	201 ± 22	194 ± 19	197 ± 19	195 ± 15

Data are presented as mean ± SD (*n* = 7 mice/group). Different superscript letters (^a,b^) within a row indicate statistically significant differences (*p* < 0.05). Mice received daily oral supplementation for 4 weeks with vehicle (glucose water), PC (2050 mg/kg/day commercial BCAAs), GU-1X (2050 mg/kg/day GU), GU-2X (4100 mg/kg/day GU), or GU-3X (6150 mg/kg/day GU). An exercise performance test was conducted 1 h after the administered dose.

**Table 3:** PERMANOVA results comparing microbial community composition between treatment groups.

Comparison	*F*	*R^2^*	*p*-Value
Vehicle vs. PC	0.7531	0.1115	1.0000
Vehicle vs. GU-1X	1.1679	0.1629	0.1880
Vehicle vs. GU-2X	1.0627	0.1505	0.3660
Vehicle vs. GU-3X	0.7506	0.2259	0.0210

*F* represents the ratio of between-group to within-group variance. *R*^2^ indicates the proportion of total variation explained by group differences. *p*-values were calculated using permutation testing; values < 0.05 indicate statistical significance.

## Data Availability

The original contributions presented in this study are included in the article. Further inquiries can be directed to the corresponding author.

## Abbreviations

The following abbreviations are used in this manuscript:

ANOVA	Analysis of variance
ASV	Amplicon sequence variants
BCAA	Branched-chain amino acid
CK	Creatine kinase
CPCoA	Constrained principal coordinates analysis
EFP	Epididymal fat pad
FFM	Fat-free mass
FM	Fat mass
GBH	Grouper bone hydrolysate
H&E	Hematoxylin and eosin
HDL	High-density lipoprotein
HED	Human-equivalent dose
IACUC	Institutional Animal Care and Use Committee
LDL	Low-density lipoprotein
NGS	Next generation sequencing
PC	Positive control
SCFA	Short-chain fatty acids
SD	Standard deviation
TC	Total cholesterol
TP	Total protein
